# Hospital Meetings

**Published:** 1904-03-26

**Authors:** 


### HOSPITAL ADMINISTRATION.

### CONSTRUCTION AND ECONOMICS.

### HOSPITAL MEETINGS.

### UNIVERSITY COLLEGE HOSPITAL.

The annual general meeting of the governors of this hospital was held on Friday, March 18th, Lord Monkswell being in the chair.

Lord Monkswell said that his first duty was to move a sincere vote of condolence to the King and the family of H.R.H. the late Duke of Cambridge. The hospital had lost a very good friend in the late Duke, who had been vice-patron since 1848, when he succeeded the Duke of Sussex, and had presided at their annual festival dinner on no fewer than three occasions. The resolution he had to propose was :—

"The governors and committee of University College Hospital have heard with profound regret of the death of H.R.H. the Duke of Cambridge, K.G., who held the office of vice-patron of the hospital from the year 1848, and also record their respectful sympathy with the members of his Royal Highness's family. The governors and committee have also to record their gratitude for the valuable help rendered to the charity by his Royal Highness in presiding upon three occasions at festival dinners in aid of the funds."

The resolution was seconded by Mr. H. Lucas and passed.

Donation of £100,000.

The Chairman then said he had to call their attention to the magnificent gift by Sir Donald Currie of £100,000, partly for the direct use of the hospital and partly to enable University College to effect amalgamation with the London University. As regarded the hospital, they were directly interested in the splendid gift of £20,000 towards building a Nurses' Home, and they were to receive further a sum of £2,500 from his three daughters—Mrs. Mirrielees, Mrs. Molteno, and Mrs. Wisely—towards furnishing the Nurses' Home. They were deeply indebted to Sir Donald Currie and his daughters for their most munificent gifts, which had come at a time when they were extremely welcome. The committee were most anxious to proceed with the building of the Nurses' Home, and this gift would be a great relief to the finances of the hospital. At the same time he hoped it would be remembered that there would still be a large deficit, particularly when the hospital was finished, with its 300 beds, and he hoped that the charitable public would assist them to the utmost extent of their power.

Vote of Thanks to Sir Donald Currie.

He then proposed a vote of thanks to Sir Donald Currie and his daughters, which was seconded by Mr. H. Lucas, who said that the hospital was interested in the whole gift, as the result of it would be that they would form part of a corporation including both the hospital and medical school. They would now be able to have a proper building for the accommodation of the medical school.

Lord Monkswell, in proposing the adoption of the report, alluded to the great loss the hospital had sustained in the death of Sir J. Blundell Maple, the munificent donor of the new hospital now in course of erection. In 1896 Sir J. Blundell Maple gave an undertaking to rebuild the hospital at a cost of £120,000, but that amount had been far exceeded, and he had made liberal provision in his will for the completion of the building, which it was confidently hoped would be completed and ready for occupation by the end of the year. The donations during the past year showed an increase as compared with the previous year. The sum of £3,000 had been received from King Edward's Fund, of which £2,000 was a special donation for the payment of tradesmen's bills. The Hospital Sunday Fund had given them £1,795, and the Saturday Fund £388. Legacies during the year amounted to over £10,000. The committee were glad to be able to announce that the expenditure had decreased by £1,122, but this was partly due to the fact that the linen had been renewed in the previous year. The *x*-ray department of the hospital has been very active during the year, no fewer than 1,122 applications of the rays having been made. The number of in-patients treated during 1903 was 2,558, out-patients and casualties 47,215, and the attendances by out-patients amounted to 165,253. The committee made a very earnest appeal for increased support to maintain the new and enlarged hospital, and pointed to the fact that while the present maintenance of the hospital cost £22,000 a year, the only reliable income was £9,000.

Mr. H. Lucas seconded the motion, which was then carried.

A vote of thanks to the Chairman terminated the proceedings.

### NATIONAL HOSPITAL FOR THE PARALYSED AND EPILEPTIC.

The first general meeting of the governors of this hospital since its incorporation by Royal Charter took place on Tuesday, the 22nd inst., Mr. J. Danvers Power Chairman of the Board of Management, presiding. Among those present were Sir John Paget, K.C., Sir Felix Semon, Lady Russell Reynolds, and several members of the Ladies' Committee.

The Chairman, in proposing the adoption of the report and accounts for 1903, referred to the severe loss sustained by the Board consequent upon the removal of Dr. Wace, now Dean of Canterbury, from London. Dr. Wace, he said, had so ably directed the affairs of the hospital during a crisis which demanded, not only tact, but also wide experience in hospital affairs that the labours of his successor in office were rendered comparatively easy. The governors present would be asked later on in the proceedings to accord their late Chairman a vote of thanks for his valuable services to the hospital during his term of office and for his continued interest in its prosperity. His Majesty the King, the Chairman continued, had been pleased to grant the royal charter of incorporation applied for since the annual meeting held in March 1903, and had also become a patron of the hospital. In connection with the charter the thanks of the committee were due to Mr. W. W. Paine, hon. solicitor, for the care and trouble taken by him in the preparation of the necessary documents: these, having been presented by him gratuitously to the hospital, represented a handsome subscription to the funds, only the necessary fees to the Privy Council having been charged to the institution. The Board, Mr. Power reminded the governors, were working under a mandate from them to carry out the recommendations of Sir Edward Fry's Committee of Enquiry, and he was glad to be able to announce that the accommodation for the nursing and domestic staff had been satisfactorily dealt with during the year, the house in Powis Place having been converted into a nursing home and connected by a passage with the hospital. With regard to the operating theatre everyone must admit that the present theatre, being constructed to perform the duty also of a lecture-room, was not in accord with the requirements of modern surgery; and it had been decided that a new one must be built forthwith. This new theatre would, he hoped, be completed in the course of the autumn, and the Board had every reason to believe that it would be opened by H.R.H. the Duchess of Albany, who had already done so much for the hospital. The grant of £500 towards improvements from King Edward's Hospital Fund had been allocated to the cost of this building, the expense of which would be something like £1,500. He had no doubt that the grants received from time to time from the King's Fund would eventually cover the cost, and in the meantime the money would be advanced from capital. He regretted that the committee could not yet see their way to providing the additional dining-room accommodation recommended by the Committee of Enquiry, while, with regard to the two houses adjoining the hospital, it had been determined to let them for 21 years provided that the lessee would spend £2,000 on them, and with the option on the part of the hospital of terminating the lease at the end of seven or fourteen years on payment of a fine. A responsible tenant had been secured on these terms, and thus, should the hospital be in a position to extend in a few years' time, it would be able to do so. The attention of the Board having been called to the occasional difficulty in filling the children's (Albany) ward, as well as to the liability to outbreaks of infectious disease among the children, and also to the need for additional male accommodation, a re-arrangement of the wards had been effected, the cots being distributed in the various wards: this had been found satisfactory, and with the governors' approval, would be made permanent.

A joint committee of representatives of the board of the medical staff had been appointed in December 1903 to consider the teaching of massage and electrical treatment, and, as a result of its report, arrangements had been made to open shortly a school of massage and electrical treatment. It was hoped that this school would become the recognised centre for teaching the Swedish system, and that it would prove a source of revenue to the hospital. Recognising that research into the origin of nervous diseases was one of the objects of the hospital, the Board had promoted an appeal for funds for carrying out this object more fully. The appeal had been commended by a number of distinguished men, among whom were Lord Lister, the President of the Royal Society, Lord Avebury, Lord Strathcona and Mount Royal, Sir Norman Lockyer, the Right Hon. Joseph Chamberlain, M.P., and the Bishop of London, in addition to the eminent scientific men associated with the hospital. He was glad to say that in response to that appeal a sufficient sum had been collected to cover a year's expenses in that department, while additions to the fund were promised, which would allow for extension of this necessary work, and consequently add to the usefulness of the hospital. With regard to the efficiency of the nursing and general conduct of the institution, a letter from Mr. J. A. Ormerod, hon. secretary of the medical committee, which had been incorporated in the report, gave ample evidence that the work was being carried on in a satisfactory manner.

Turning to the gloomy subject of finance," the Chairman said the committee had every reason to congratulate themselves on the details of their financial position, but he was sorry to say that the net result was exceedingly unsatisfactory. Although after an extremely bad year the subscription list had been kept to within £3 of that for 1903, and donations showed an increase of £1,268 19s., legacies amounted only to £2,389 for general purposes, as against £6,568 in 1902, while the ordinary expenditure amounted to £19,647, as against £18,660 in the previous year, the consequence being that a sum of £6,124 had been withdrawn from capital in order to meet current expenses. In this connection he decided to mention the appointment of two committees, one consisting of ladies, to enquire into the domestic expenditure of the establishment, including provisions, and the other to examine the prices and expenditure on drugs, etc. The Board had reason to believe that the ladies' committee had effected a saving of over £300 a year, and the drugs committee of £50 a year. In accordance with the recommendation of the first-mentioned committee, the office of steward had been abolished, and a housekeeper had been appointed. This effected also the saving of the organist's salary, since the housekeeper was competent to discharge the duties of that office. It would, he added, be an injustice to their excellent secretary, Mr. G. H. Hamilton, if he were not to place on record the indebtedness of the hospital to him for his successful efforts to add to the income of the hospital; it was greatly to his honour that it had attained the figures at which it stood last year. Neither could he omit to mention the success attending the festival dinner of April last, resulting in a net gain of £1,611 to the funds. The Board desired to acknowledge their indebtedness to Sir Marcus Samuel, who presided, and who, having made himself personally acquainted with the needs of the hospital, commended the charity to the public in an admirable speech. Mr. Danvers-Power concluded with a warm tribute to the value of the presence on the Board of the medical representatives, whose regular attendance at meetings, together with their practical advice and help in administration, were of the greatest possible advantage. To Miss Vernet also he wished to express their thanks for the continued excellence of the nursing department of the hospital.

Dr. Beaver having seconded the motion, and some criticisms having been made on several points in the report by Mr. Burford Rawlings, Sir Felix Semon and the Chairman invited that gentleman to give the hospital the benefit of his unique experience in hospital finance, thus assisting the Board in their endeavour to keep down the expenditure and to add to the permanent source of income.

The motion was then carried.

The Earl of Dudley having been re-elected President for the ensuing year, a vote of thanks to Sir Felix Semon, retiring surgeon, for his distinguished services to the hospital was passed, and other business having been transacted the proceedings terminated.

### THE ROYAL EYE HOSPITAL.

THE annual meeting of this institution was held on Wednesday, March 16th, Mr. W. Moresby-Chinnery, President, in the chair.

The report and accounts for 1903 having been adopted, Professor M. McHardy said the need of the hospital was a new out-patients' department. When the present premises were designed in 1889, the number of new out-patients attending was only from 5,000 to 6,000, and accommodation was provided for these to increase three or four fold. The hospital, therefore, was now capable of dealing with 20,000 new out-patients annually, and for that number it had an almost adequate provision of beds. The full limit had, since the opening of the new century, been reached and passed, and the resulting overcrowding and inconvenience had been forcibly brought to the attention of the Council by the medical officers, by patients or their friends, and by the officials of the Hospital Saturday Fund. In 1903 the new out-patients numbered 23,295, exclusive of 650 admitted for in-patient treatment, and of 2,113 accident and urgent casualty cases, and to meet the increase the premises had been opened both morning and afternoon. But even so, with a double shift of officers, the department was dangerously overcrowded. Since 1899, when the necessity for extending the charity was recognised, the Council had been arranging for an extension of the site, and they now appealed for generous contributions to the building and extension fund, since the outlay incidental to the enlargement of the department alluded to must be immediately incurred. It would, he added, be economical to anticipate the aggravated demand that would arise for beds by providing additional ward, nursing, and domestic accommodation. The work of the out-patients' department, he continued, had been rendered more congested than it would otherwise have been by the influx of large numbers of children from the elementary schools, the testing of whose sight, while requiring very careful attention, could not be permitted to take precedence of urgent accident and casual cases. The hospital had incurred some amount of blame on the part of the school authorities on this account, but while fully admitting the national value of that branch of ophthalmic work, he submitted that it added very greatly to the gratuitous labours of the staff. In reply to a question put by one of the governors present, Mr. McHardy said the amount required for the extension was £8,000, of which some £1,200 was already in hand.

### THE LONDON TEMPERANCE HOSPITAL.

The usual anniversary meetings of the London Temperance Hospital took place on Thursday, March 17th.

At the governors' meeting in the afternoon, under the chairmanship of Mr. Alderman Strong, J.P., the annual report and accounts were adopted; Sir George Livesey was re-elected President for the current year, Mr. H. Holloway was re-elected Treasurer, and Prebendary Russell Wakefield (Mayor of St. Marylebone) and the Rev. Canon Barker were elected to the Board of Management. Other routine business having been transacted, the usual votes of thanks were passed, and at the close of the meeting the Matron (Miss Richardson) was presented by the committee with a gold medal in recognition of her services to the hospital during the last 13 years.

At the evening meeting, at which a large number of friends and supporters of the hospital were present, the chair was taken by Sir George Livesey, who was warmly received. There were also on the platform the Rev. V. G. Borradaile, Dr. Dawson Burns, the Rev. C. Silvester Horne, Alderman Strong (Chairman of the Board of Management), and others. Letters of regret had been received from the Archbishop of Canterbury and from Earl Roberts, who wrote that he had read the annual report with great interest, and was pleased to note the splendid work accomplished during the year.

The first resolution was moved by the Rev. C. S. Horne, to the effect that "the practice of this hospital in the treatment of medical and surgical cases without the ordinary use of alcohol, having been fully justified by an experience extending over 30 years, affords a guiding light to the medical reformer and strengthens the scientific lessons of the temperance reformation. This meeting therefore appeals to the friends of enlightened philanthropy to render such generous assistance as will enable the usefulness of the institution to be greatly and permanently augmented." Every fair-minded critic, said Mr. Horne, must be convinced that the hospital had passed the experimental stage, and had successfully demonstrated the scientific theory with which it set out, namely, that stimulants were not necessary in the treatment of disease. He claimed, further, that it had proved to be an educational factor among the poor, to whom the idea of recovery from any illness without recourse to alcohol was formerly almost unknown. The hospital had also played its part in the very remarkable spread of temperance sentiments throughout the medical faculty of this country; and temperance reformers were proud to reckon among their supporters men like Sir William Collins, Sir Victor Horsley, and Professor Sims Woodhead.

Mr. Farraker having seconded the resolution, it was put from the chair, Sir George Livesey remarking that if people could be induced to be more thrifty and to drink less the need for institutions such as the Temperance Hospital would be considerably reduced. Sickness and suffering, however, did exist, and it was therefore incumbent upon every member of the community to help to relieve it. Enlightened philanthropy meant Christianity, and had a universal claim. This hospital was one of the grandest experiments ever made, and he earnestly commended it to the generous support of the public. The resolution was carried.

Sir William Collins responded on behalf of the visiting staff. It would have been more appropriate, he thought, if this part of the programme had been in the hands of the friends of enlightened philanthropy just alluded to, and a practical response had already been made by a visitor, who was so struck by the beauty of the aseptic ward that he wished immediately to write a cheque for the hospital. The chairman was an advocate of profit-sharing; he (the speaker) would venture to suggest that the needs of this hospital afforded opportunities for profit-sharing in another sense by those who had been thrifty enough to lay by in the past. The hospital, he went on to say, had concluded another year of satisfactory progress, and continued to demonstrate the soundness of the principles upon which it was based. The laity, he cordially admitted, had in this case led the profession, and they had their justification in the scientific results which the profession was able to place before the public to-day. The question which Sir James Paget used to say should be asked in regard to every practice, viz. "What would happen if this were not done?" had been asked and answered, and the medical press, he recognised with pleasure, was pervaded by a far more friendly tone than used formerly to be the case —a clear proof that the idea exemplified by that hospital was gaining ground. The hospital endeavoured to keep abreast of scientific reform, and the ward to which he had already alluded stood for a model of its kind, and had been copied, in plan and construction, by the Admiralty after a visit of inspection. But unless this institution was to be out-distanced by its friendly rivals in London, there was one department at least which must be brought into line, and that without delay—he alluded to the out-patients' department. This was at present conducted in the old vicarage adjoining the neighbouring church, with an iron annex, and a new building was an urgent necessity to cope with the large numbers applying for treatment, viz. 22,937 during 1903. These figures included 14,524 casualty cases, and the total number of visits was 62,377. He earnestly appealed for the £10,000 necessary to build this most urgently needed adjunct. If a larger sum were forthcoming, the surplus might well be employed in providing a convalescent home. The operating theatre, also, was out of date, and needed to be renovated, while he was also strongly of opinion that adequate laboratories should be associated with the new out-patients' department. He concluded by expressing his thanks to the resident staff, including the matron and nurses, for their admirable services to the hospital during the year.

Dr. Dawson Burns having replied on behalf of the Board of Management, Mr. Alderman Strong endorsed the remarks made by Sir William Collins with regard to the out-patients' department. The erection of new premises was imperative, and a condition had been made by the representatives of King Edward's Fund that it should be begun before the end of the present year. The sum of £2,000 had been contributed by that fund for the purpose, and it now rested with the subscribers to aid the committee to carry out the scheme which had already been inaugurated. Although the statistics given in the reports for recent years showed a slight falling-off in the numbers of out-patients, he believed the depression was accountable for, being due to the rebuilding of a neighbouring hospital, that hospital being now re-opened, their numbers had merely reverted, and stood at an average of 200 for every working day during the last two years. The condition alluded to, which accompanied the last grant of £500 from King Edward's Fund, showed that this independent body of experts was becoming impatient. The sum of £500 was realised at a meeting in October, under the auspices of the then Lord Mayor; there remained, therefore, £7,500 to be raised. The committee were perfectly convinced of the necessity for this outlay, and appealed to the subscribers to support them in their desire to bring this department into line with the rest of the building.

The meeting closed with votes of thanks.

### METROPOLITAN CONVALESCENT INSTITUTION.

The annual meeting of the Governors of the Metropolitan Convalescent Institution was held at the Office, 32 Sackville Street, W., on the 8th inst., Mr. M. O. Fitz-Gerald in the Chair.

The sixty-fourth annual report of the Board of Management stated that during 1903 no fewer than 6,893 men, women and children were admitted to the Homes situated at Walton, Bexhill, St. Leonards and Broadstairs, a larger number by 600 than in any previous year. The income of the Charity was £13,163 and the expenditure £13,657. Reference was made in the report to the financial position of the Charity and to the fact that the Walton and Broadstairs Homes, which have hitherto had one general fund, would be maintained by separate funds in future; and it was also announced that certain alterations had been made in the privileges of donors and subscribers.

A new home for men only is in course of erection at Little Common, Bexhill, which it is hoped will be ready for occupation next Spring. The cost of the Home when completed—a portion only is being built now owing to want of funds—will be about £22,000, and an appeal is made for £10,000 to enable the Governors to put the whole work in hand at once.

Thanks were accorded to the Honorary Physicians and Surgeons and the other officers, including Messrs. Price, Waterhouse and Company, the auditors, for their services during the year, and other formal business having been transacted the proceedings terminated.

